# Study on the Anti-Biodegradation Property of Tunicate Cellulose

**DOI:** 10.3390/polym12123071

**Published:** 2020-12-21

**Authors:** Yanan Cheng, Ajoy Kanti Mondal, Shuai Wu, Dezhong Xu, Dengwen Ning, Yonghao Ni, Fang Huang

**Affiliations:** 1 College of Material Engineering, Fujian Agriculture and Forestry University, Fuzhou 350108, China; yanan.cheng@fafu.edu.cn (Y.C.); ajoymondal325@gmail.com (A.K.M.); dmxj0609@163.com (S.W.); xudezhong95@163.com (D.X.); dengwen-ning@m.fafu.edu.cn (D.N.); 2 Institute of Fuel Research and Development, Bangladesh Council of Scientific and Industrial Research, Dhaka 1205, Bangladesh; 3 Department of Chemical Engineering, University of New Brunswick, Fredericton, NB E3B 5A3, Canada

**Keywords:** tunicate cellulose, crystalline, enzymatic hydrolysis

## Abstract

Tunicate is a kind of marine animal, and its outer sheath consists of almost pure I*_β_* crystalline cellulose. Due to its high aspect ratio, tunicate cellulose has excellent physical properties. It draws extensive attention in the construction of robust functional materials. However, there is little research on its biological activity. In this study, cellulose enzymatic hydrolysis was conducted on tunicate cellulose. During the hydrolysis, the crystalline behaviors, i.e., crystallinity index (*CrI*), crystalline size and degree of polymerization (DP), were analyzed on the tunicate cellulose. As comparisons, similar hydrolyses were performed on cellulose samples with relatively low *CrI*, namely α-cellulose and amorphous cellulose. The results showed that the *CrI* of tunicate cellulose and α-cellulose was 93.9% and 70.9%, respectively; and after 96 h of hydrolysis, the crystallinity, crystalline size and DP remained constant on the tunicate cellulose, and the cellulose conversion rate was below 7.8%. While the crystalline structure of α-cellulose was significantly damaged and the cellulose conversion rate exceeded 83.8% at the end of 72 h hydrolysis, the amorphous cellulose was completely converted to glucose after 7 h hydrolysis, and the DP decreased about 27.9%. In addition, tunicate cellulose has high anti-mold abilities, owing to its highly crystalized I*_β_* lattice. It can be concluded that tunicate cellulose has significant resistance to enzymatic hydrolysis and could be potentially applied as anti-biodegradation materials.

## 1. Introduction

Cellulose is the most widely distributed, abundant and renewable polymer in nature. In the cellulose-degradation ecosystem, many cellulose-decomposing bacteria and fungi could hydrolyze insoluble cellulose substrates into soluble sugars, mainly cellobiose and glucose, which are then absorbed by cells. Generally, these microorganisms can produce various enzymes (i.e., cellulase), to catalyze the cellulose degradation. Cellulase is the general name of a group of enzymes, namely the endoglucanase, exogenous glucosidase and cellobiose. The synergistic effects of these enzymes induce the degradation of cellulose into monosugars.

Structural studies of cellulose show that cellulose is a homogeneous polycrystalline macromolecule compound. It contains crystalline and amorphous regions. In the crystalline region, the arrangement of cellulose molecules is completely regular. The connection between crystalline regions is amorphous regions, and there is no obvious boundary between crystalline and amorphous regions. The ratio of crystallization zone to the amorphous zone varies with the fiber types and the fiber locations. The physical and chemical properties of cellulose are closely related to its crystalline structures. Generally, the completely disordered amorphous region is more susceptible to the enzymatic hydrolysis than the regular crystalline regions. Therefore, understanding the crystallinity and morphology of cells plays an important role in enzymatic hydrolysis.

Cellulose can be found in plants (e.g., cotton, hemp and wood), marine animals (e.g., tunicates), algae (e.g., *Valonia*), bacteria (e.g., *Acetobacterxylium*) and even amoeba (*Dictyosteliumdiscoideum*) [1]. Even though they have the same chemical composition, their crystalline structures are significantly different, rendering them different physical and chemical properties. The tunicate cellulose is the only cellulose found so far from animals that is mainly in its capsule. The tough capsule mostly composed of cellulose and a small amount of proteins and inorganic compounds protecting the soft organs of the tunicate [2]. The tunicate cellulose is almost pure I*_β_* crystalline cellulose. It has a unique monoclinic lattice, rendering it thermodynamically more stable than the triclinic lattice in cellulose I*_α_*. In addition, the tunicate cellulose crystal has an exceptionally high (>70) length-to-diameter ratio (i.e., aspect ratio), indicating excellent mechanical properties [3].

Mainly due to its unique physical structures and chemical properties, intensive research has explored its applications on electrical- [4,5,6,7] and thermal-conductive [8], optical [9,10], and medical materials [11,12]. Furthermore, several studies were done to utilize cellulosic nanofibers in various fields, including the following: to remove heavy metal ions and degrade dye from water [13,14,15]; as an electrochemical energy storage devices [15,16,17]; as reinforced polymer composites [15]; and as cosmetic products, wound dressings, drug carriers, medical implants, tissue engineering, food and composites [15,18].

However, there is little research in the literature on the biological activity of tunicate cellulose. In this study, the enzymatic hydrolysis and anti-mold experiments were conducted on tunicate cellulose. During the hydrolysis, the variations of cellulose crystallinity, crystal size and degree of polymerization were extensively evaluated. As comparisons, similar hydrolysis were performed on cellulose samples with a relatively low crystallinity index (*CrI*), namely α-cellulose and amorphous cellulose. In the anti-mold experiments, different percentages of tunicate nanocrystals were mixed in the cultivated medium, and the mold growth rates were monitored. The objective of this research was to establish the relationship between the tunicate cellulose crystalline structure and its anti-biodegradation properties. As far as we know, this is the first research on the biodegradation properties of marine animal (tunicate) cellulose. This research should surely be helpful to extend the application of tunicate cellulose in the biological domain.

## 2. Materials and Methods

### 2.1. Materials

Red reef tunicates (*Rhopalaeaabdominalis*) were purchased from Weihai Sea Food Market in Shandong, China. The mold strain (*Aspergillusniger*) and the modified Martin agar medium were purchased from DATA Laboratory Equipment Reagent (Tianjin, China). The cellulose enzyme was kindly donated by Vland Biotech (Qingdao, China). The radiate pine dissolving pulp was donated by Qingshan Paper (Fujian, China). All the chemicals used in this study, including KOH, KBr, HCl, H_2_SO_4_, NaClO_2_, CH_3_ONa, CH_3_OH, C_3_H_7_OH, uranyl acetate and acetate buffer, were of analytical grade and were procured from Beijing Inno Chem Science & Technology Co. Ltd (Beijing, China).

### 2.2. Methods

#### 2.2.1. Preparation of Tunicate Cellulose

The tunicate specimens were slit open with the help of a sharp knife, and their mantles were washed thoroughly under deionized water. Whole mantles were soaked for a whole night, in a 5 (% *w*/*v*) aqueous KOH solution, at normal temperature. The mantles were then rinsed with DI water and bleached for 6 h at 70 °C, with a bleaching solution, exchanging the used bleaching solution with a fresh one every 2 h. Then, 300 mL 2% (6 g) chlorite solution and 5 mL of anhydrous (water-free) acetic acid were mixed properly, to make bleaching solution. These treatments, i.e., the adding of KOH, followed by bleaching, were repeated four times, until the mantles became completely white. At last they were washed thoroughly and cut into small pieces, for further characterizations.

#### 2.2.2. Preparation of Amorphous Cellulose

Purified fibrous cotton hydrocellulose (1.0 g) and DMSO (100 mL) were mixed by stirring at 125 °C. Then paraformaldehyde (5.0 g) was added into the mixture, and stirring was carried on until a clear solution was acquired, and the methyl cellulose solution was diluted with DMSO (400 mL) and allowed to cool down at normal temperature. After that, the solution was filtered through 1.2 μm glass microfiber. The solution of 0.2 mol/L sodium alkoxide in methanol:2-propanol (1:1, vol: 2 L) was added, dropwise, to the stirred regeneration bath. The cellulose precipitate was washed with 0.2 mol/L sodium alkoxide in methanol:2-propanol (1:1, vol: 600 mL), methanol (3 × 600 mL), 0.1 mol/L HCl (600 mL), distilled water (3 × 600 mL), 0.1 mol/L HCl (600 mL) and distilled water (3 × 600 mL) and then freeze-dried prior to use.

#### 2.2.3. Preparation of Softwood α-Cellulose

The α-cellulose was prepared from the dissolving pulp, following the literature method [19]. At first, pulp samples were treated with 17.5% NaOH solution at 20 °C for 45 min, to remove the hemicellulose. Most of the non-cellulosic carbohydrates were dissolved in the basic solution, and the cellulose was insoluble. The solid residue was washed with DI water, until neutral. The retained solid cellulose was denoted as α-cellulose.

#### 2.2.4. Enzymatic Hydrolysis of Cellulose Samples

Cellulose samples were hydrolyzed by using a commercial enzyme (cellulase and cellobiose). The activity of cellulase was 103.3 FPU determined by the NREL method [20]. Enzymatic hydrolysis of cellulose substrates was carried on at 5% (*w*/*v*), in 50 mL of 50 mM acetate buffer (pH 4.8), in an incubator shaker (IS-RDH1, Jiangsu Taicang equipment factory, China), at 45 °C, with rotation speed of 150 rpm. The substrate was put on the incubation shaker for 10 min with buffer mixtures, following the adding of enzymes. Enzymatic hydrolysis was halted by boiling the sample for 5 min, to inactivate the enzyme activities. Liquid samples were extracted and filtered at various time intervals, for the sugar-release analysis. All of the extracted liquid samples were further analyzed for glucose content by high-performance ion-exchange chromatography with pulsed amperometric detection (HPAEC-PAD, Dionex IC 3000, USA). The cellulose conversion rate (%) was calculated as Equation (1) [21]:

(1) $$Cellulose\;conversion\;rate=\frac{0.9\times 100\times glucose\;\left(g\right)}{Initial\;cellulose\;\left(g\right)}$$

The solid residue (non-hydrolyzed solid) was extensively washed with DI water, to remove non-adsorbed enzymes. After that, the samples were air-dried for the XRD analysis.

#### 2.2.5. Characterization

##### XRD

The crystallinity was evaluated by using the XRD patterns that were recorded by X-ray diffractometer Rigaku RINT 2200 equipped with monochromator, which is an important method to study the ultrafine properties of cellulose. It is the most direct method for analyzing the cellulose crystalline structure and crystal orientations. X-ray diffraction was conducted on reflectance modes through 7.5° < 2θ < 32.5° by Cu–Kα radiation, operated at 40 kV and 30 mA. The cellulose crystallinity was measured by deconvolution method as previously reported [22]. The cellulose crystallization index, crystallinity and crystal size were characterized based on X-ray diffraction patterns and peak-splitting results.

The crystallization index (*CrI*) was calculated according to Segal [23] Equation (2).

(2) $$CrI=\frac{I_{cr}-I_{am}}{I_{cr}}\times 100\%$$

where *I_cr_* is maximum diffraction intensity of 002 surface (for cellulose I) or 101 surface (for cellulose II), and *I_am_* is diffraction intensity in amorphous region.

The crystal size can be calculated according to Scherer [24] method, according to Equation (3).

(3) $$I_{hkI}=\frac{K\lambda }{\beta \cos\theta }$$

where *I_hkl_* is the crystal size, *K* is the *Sherrow* constant (*K* = 0.9, *λ* = 0.1542 nm) and *β* is the maximum half-width of the characteristic diffraction peak, expressed in radian.

##### Degree of Polymerization

Gel permeation chromatography (GPC) (Waters, Polymer Standards Service, Milford, CT, USA) was used to determine the weight average molecular weight (*M_w_*) distribution and degree of polymerization (DP) of cellulose samples. At first, the cellulose samples were derivatized by phenyl isocyanate according to the published literature method [25,26]. From each sample, 15.0 mg of cellulose was taken in test tubes outfitted with micro stir bars, and it was vacuum-dried overnight, at 40 °C. Then anhydrous pyridine (4.00 mL) and phenyl isocyanate (0.50 mL) were added, and the test tubes were capped properly with rubber septa. To complete the reaction, the test tubes were kept in an oil bath, with continuing stirring, for 72 h. The reaction was stopped by adding of 1.00 mL of CH_3_OH, and, later, the resulting mixture was added dropwise to a 7:3 CH_3_OH/H_2_O solution (100.0 mL), so that the precipitation of derivatized cellulose could be promoted. Solid products were collected by filtration and thoroughly washed with CH_3_OH/H_2_O solution (1 × 50.0 mL) and later with H_2_O (2 × 50.0 mL). Finely washed derivatized cellulose was dried overnight, under vacuum, at 40 °C. GPC analysis was performed by dissolving the derivatized cellulose in tetrahydrofuran (2 mg/mL), followed by filtering through a 0.22 μm filter and then being placed in a 2 mL auto-sampler vial.

##### FTIR Analysis

The FTIR spectra were acquired by using the KBr pellet technique. A very tiny amount of sample was mixed with KBr (1 mg sample/100 mg KBr) and grinded in an agate mortar. A pellet of the grinding mixture was prepared by pressing with hydraulic pressure. Spectra were registered by using the VERTEX 70 spectrometer (Bruker, Billerica, MA, USA), at the range from 4000 to 450 cm^−1^, at a resolution of 4 cm^−1^.

#### 2.2.6. Anti-Mold Experiment

In order to perform the anti-mold experiment, the substrate should be evenly dispersed in the agar medium. The tunicate cellulose prepared in Section 2.2.2 was in the form of flakes, while the softwood α-cellulose prepared in Section 2.2.3 was in big particles. Evidently, it is difficult to disperse these two celluloses in the cultivated medium. To solve this problem, the tunicate cellulose and softwood α-cellulose were hydrolyzed to cellulose nanocrystals by H_2_SO_4_, as described in the preparation of cellulose nanocrystals. In addition, XRD analysis was performed on these cellulose nanocrystals, with the aim of checking the *CrI* changes during the acid hydrolysis. The anti-mold experiments were only conducted on the tunicate and softwood nanocrystals. The amorphous cellulose was excluded from the experiment because of its poor anti-biodegradations.

##### Preparation of Cellulose Nanocrystals

Cellulose nanocrystals were prepared by acid hydrolysis process of softwood pulp, following the literature method [27]. In brief, 60.0 g of oven dried pulp was mixed with H_2_SO_4_ (64%, *w*/*w*, 1:10 g/mL) solution at 45 °C and stirring continuously for about 45 min. An excess amount (10-fold) of distilled water was added in the solution, to stop the hydrolysis reaction. To remove acid and water from the solution, it was centrifuged at 12,000 rpm for 10 min, until the supernatant became turbid. The sediment containing nanocellulose was collected and dialyzed (MWCO: 12–14,000) against tap water, until the solution pH became neutral. The content after dialysis was sonicated for 10 min and centrifuged again, at 10,000 rpm, for 5 min. The cloudy supernatant, containing nanocellulose, was collected, and the remaining sediment was again mixed with water, sonicated and centrifuged to obtain extra nanocellulose, and this process was repeated till the supernatant was clear.

##### TEM Analysis of Prepared Softwood Cellulose Nanocrystal

Transmission electron microscopy (TEM) was used to measure the dimensions of the prepared cellulose nanocrystals. From the nanocrystal suspension (1 wt%), one drop was accumulated on the surface of a tidy copper grid covered with a porous thin carbon film. A drop of a diluted suspension was deposited on the surface of a clean copper grid covered with a porous thin carbon film. The samples were then stained by allowing the grids to float in a 2 wt% solution of uranyl acetate for 3 min. At last, the samples were dried at room temperature, for 24 h. The grid was analyzed in a TECNAI G2 F20TEM (US FEI Co.), with 0.2 nm resolution. The length and width of prepared cellulose nanocrystals were measured by using the software Gatan Digital Micrograph.

##### Anti-Mold Effect of Cellulose Nanocrystals

Prior to use, the mold (*Aspergillusniger*) should be activated in the medium. The activation was performed according to the following procedures. Briefly, the strain was inoculated onto the modified Martin agar medium and cultured in a biochemical incubator, at 26 °C, for one week [28,29]. The medium and tunicate cellulose nanocrystals were sterilized at 121 °C for 20 min. The medium and tunicate cellulose nanocrystals were thoroughly mixed according to the dry weight ratio of 0%, 10%, 30%, 50%, 80% and 100%, to prepare a flat plate. In the center of each plate, a hole of 0.7 cm in diameter was drilled to receive activated mold strain by a hole punch tool [30]. Then the activated *mold* cake with the same diameter was transferred to the hole, by using the same tool. The flat plate was then cultivated in a biochemical incubator, at 26 °C. The radius of mold growth was measured after 12, 24, 36, 48, 60 and 72 h, respectively. In order to compared the anti-mold ability of tunicate celluloses with other cellulose samples, a similar medium (50 wt%) was mixed with α-cellulose nanocrystals (50 wt%), to perform the same experiments. In addition, a blank sample was donated as the pure Martin agar medium in the cultivation.

##### Growth Rate Analysis

**Figure 1:**
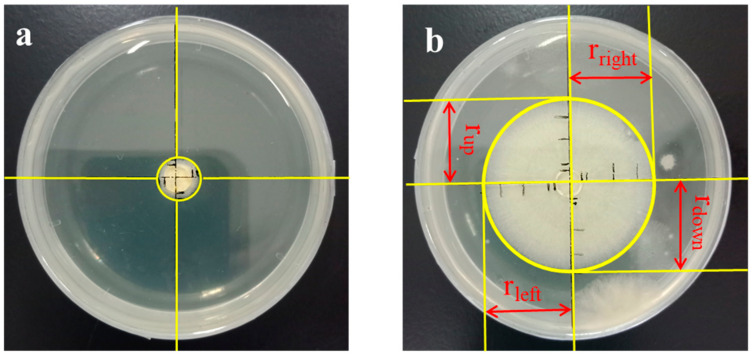
Anti-mold experiment process of tunicate cellulose: (**a**) initial growth of mold and (**b**) growth of mold at time, t.

Due to the uneven growth of mold, the radius of different mold growth was measured from the top, bottom, left and right directions (Figure 1). The growth rate of mold was calculated by the following equations.

(4) $$r=\frac{r_{top}+r_{bottom}+r_{left}+r_{down}}{4}$$

where *r_top_*, *r_bottom_*, *r_left_* and *r_right_* are the radius (mm) measured from the top, bottom, left and right directions, and “r” refers to average growth radius (mm).

(5) $$Growth\;rate=\frac{\Delta r_{t}}{\Delta t}$$

where Δ*t* is the cultivation time (h), and Δ*r_t_* is the growth radius at Δ*t* (h) time period.

### 2.3. Error Analysis

Triple measurements were conducted for all the characterization techniques, and a mean value was reported. The error margin associated with all the analysis was within ± 2.5%.

## 3. Results and Discussion

### 3.1. XRD Analysis

**Figure 2:**
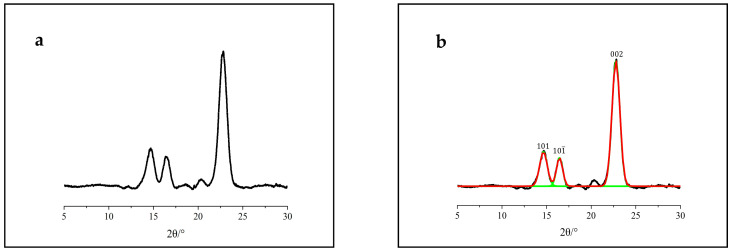
XRD patterns of tunicate cellulose: (**a**) original XRD pattern and (**b**) peak separation results.

**Figure 3:**
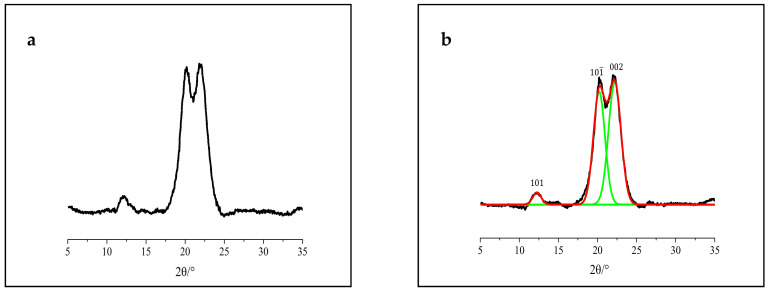
XRD patterns of α-cellulose: (**a**) original XRD patterns and (**b**) peak separation results.

**Figure 4:**
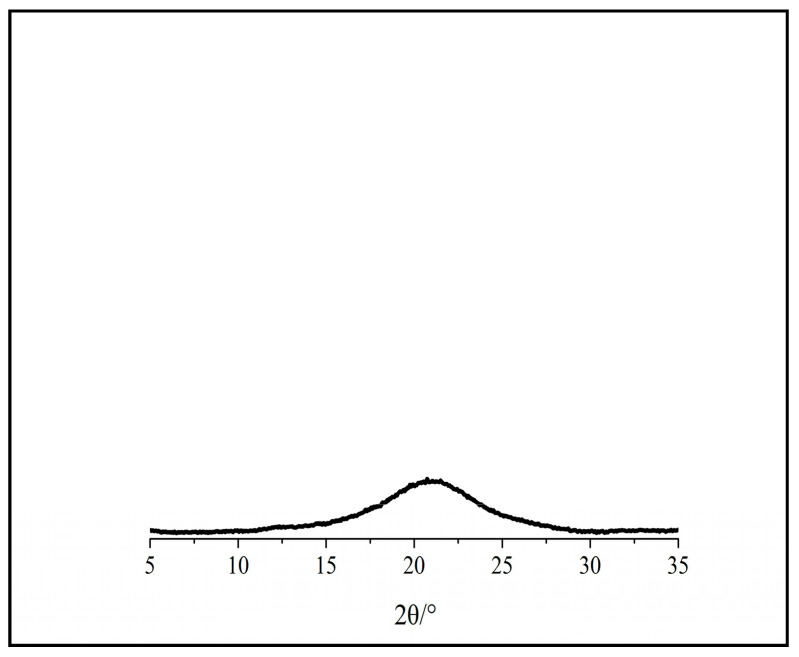
Patterns of amorphous cellulose.

Cellulosic compounds have both crystalline and amorphous region, and XRD analyses were performed to identify the crystalline and amorphous structure [31,32]. In order to systematically analyze the cellulose crystal patterns, the Gauss function in origin 9.1 software was used to separate the XRD intensity peaks, aiming to identify the cellulose crystal lattice planes. Figure 2, Figure 3 and Figure 4 represent the XRD patterns of tunicate cellulose, α-cellulose and amorphous cellulose samples, respectively. The XRD analyses on the cellulose *CrI* and *I_hkl_* (crystallite size) are listed in Table 1. Figure 2a and Figure 3a, show the original diffraction patterns of XRD, and the Figure 2b and Figure 3b represent the peak separations. It can be clearly seen that both the tunicate cellulose and α-cellulose have typical (101), (101) and (002) lattice planes in XRD patterns. Tunicate cellulose show the peak values of the position at 2θ = 22.9°, 16.6° and 14.8° for the typical (002), (101) and (101) lattice planes, respectively. These results indicate that prepared tunicate cellulose has a good crystalline structure, which is the good agreement of the published literature [33]. The XRD peak fitting indicated that the *CrI* and crystalline size (*I_hkl_*) of tunicate cellulose areas were as high as 93.9% and 9.3 nm, respectively, as shown in Table 1. These data were close to the literature report [34]. The narrow and sharp diffraction peak at 22.9° (Figure 2) also indicated the excellent crystalline structure of tunicate cellulose [35].

Figure 3 shows the α-cellulose crystal pattern, and the peaks value at the position of (101), (101) and (002) lattice planes are 2θ = 12.1°, 20.2° and 22.1°, respectively. Moreover, α-cellulose is semi-crystalline in nature. The peak values at the position of 2θ = 20.2° and 15.0° represent the crystalline and amorphous structure, respectively [36,37]. Similar results were reported for the characterization of α-cellulose [38]. The XRD peak fitting of *CrI* and crystalline size (*I_hkl_*) for α-cellulose is 70.9% and 2.1 nm, respectively, as shown in Table 1. These measurements were in agreement with the literature data [34].

Figure 4 demonstrates the amorphous cellulose, indicating the absence or strong reduction of all peaks corresponding to crystal lattice planes (101), (101) and (002). A broaden peak shows at 2θ = 21° region, indicating the typical amorphous cellulose structure [22,39,40]. The amorphous cellulose did not show any crystallite structures, as indicated in Table 1.

### 3.2. FTIR Analysis

**Figure 5:**
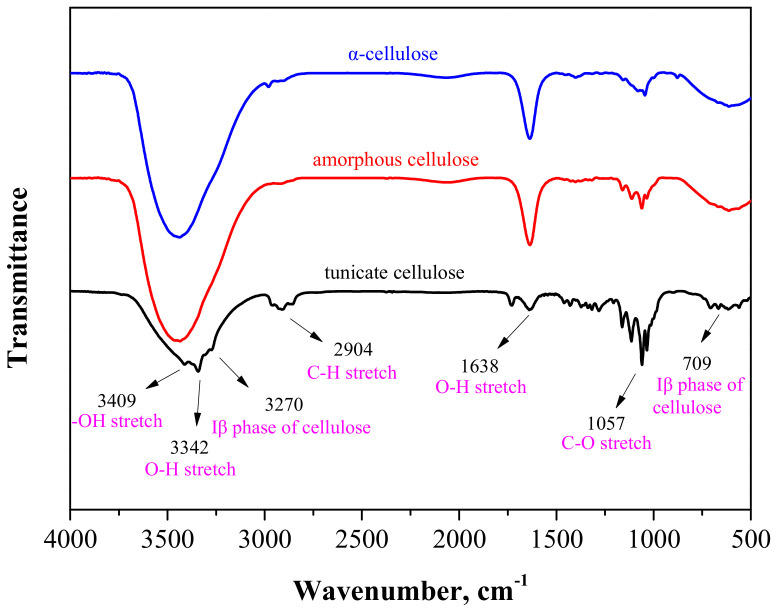
FTIR spectra of tunicate, pulp *α* and amorphous cellulose.

Figure 5 represents the FTIR spectra of α-, amorphous and tunicate celluloses. The transmittance bands are detected in two wave number regions of 3700–2500 cm^−1^ and 1750–500 cm^−1^. The transmittance band in pulp α-cellulose and amorphous cellulose was observed at the range of 3700–2900 cm^−1^, corresponding to the vibrational stretching of hydrogen bonded O-H and C-H bonds in polysaccharides. A broad transmittance band at around 3409 cm^−1^ is represented to the characteristic vibrational stretching of hydroxyl group in polysaccharides [41,42]. This band also indicates the intra- and inter-molecular hydrogen bond vibrations in cellulose [43]. The transmittance band near 2904 cm^−1^ is assigned to the C-H stretching vibration of all hydrocarbon components in polysaccharides [42,44]. Typical peaks attributed to cellulose were detected in the range of 1630–900 cm^−1^. The bands located at 1638 cm^−1^ match to O-H bending vibration of water molecules absorbed in cellulose [45]. The transmittance peaks at 1057 cm^−1^ belong to stretching vibrations of C-O and C-C bonds in cellulose [45,46].

Figure 5 illustrates that tunicate cellulose shows the typical characteristics transmittance of cellulose for the stretching vibrations of O-H, C-H and C-O at 3342, 2904 and 1057 cm^−1^, respectively [45]. Two transmittance bands near 3270 and 709 cm^−1^ in tunicate cellulose indicated the I*_β_* phase of cellulose [41], whereas these bands are absent in α-cellulose and amorphous cellulose. These two peaks clearly proved that tunicate cellulose has and I*_β_* crystalline structure.

### 3.3. XRD Analysis of Cellulose after Enzymatic Hydrolysis

**Figure 6:**
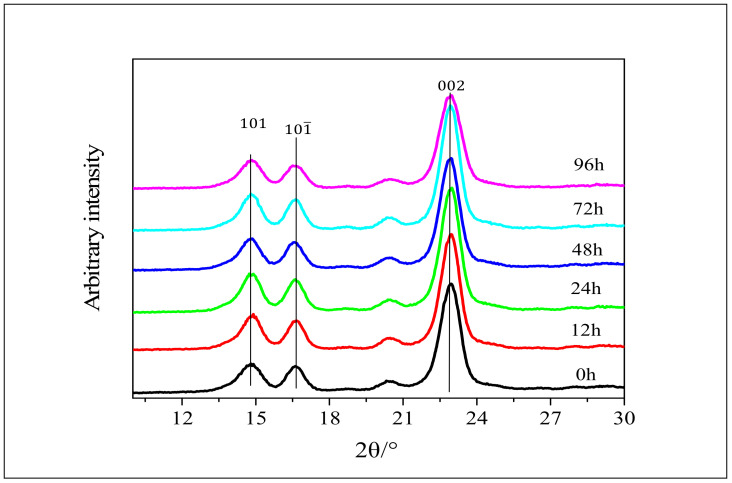
The XRD pattern of tunicate cellulose after enzymatic hydrolysis.

In order to investigate the biological activity of tunicate cellulose under the action of enzymes, enzymatic hydrolysis of tunicate cellulose, α-cellulose and amorphous cellulose was carried out. Wide-angle X-ray diffraction (WAXD) scanning was accomplished in the range of 2θ = 5~35° for the tunicate and α-celluloses. Figure 6 shows the XRD patterns of the tunicate cellulose after enzymatic hydrolysis of different time intervals (0, 12, 24, 48, 72 and 96 h). It can be seen that the intensity of 2θ = 22.9° is almost unchanged between the initial hydrolysis stage and different time intervals (0, 12, 24, 48, 72 and 96 h). The peak values of the position at 2θ = 14.8°, 16.6° and 20.3° are the same even after different hydrolysis time intervals. Tunicate cellulose has a highly crystalline structure (Figure 2), as compared to α-cellulose and amorphous cellulose, rendering it stable in enzymatic hydrolysis and causing it to retain its crystal structure up to 96 h. The crystal structure of tunicate cellulose forms a physical barrier to the enzyme, resulting its intact crystal lattice in the XRD pattern [47].

**Figure 7:**
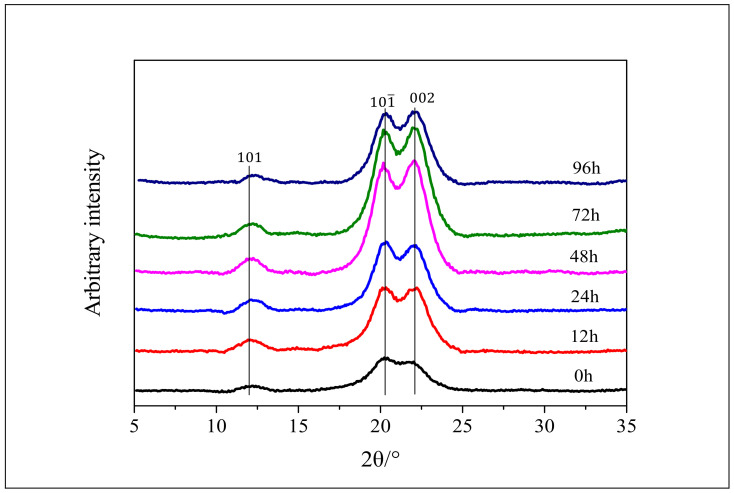
The XRD pattern of α-cellulose after enzymatic hydrolysis.

Figure 7 shows the XRD patterns of α-cellulose after enzymatic hydrolysis of different time intervals (0, 12, 24, 48, 72, 96 h), and some significant changes were observed at different stages. The strengths at the position of 2θ = 20.2° and 22.1° are gradually increased with the prolongation of hydrolysis time. Moreover, α-cellulose has both crystalline and amorphous regions in the cellulose structure. The amorphous region is unstable and is easily degraded during enzymatic hydrolysis [48]. Compared with the amorphous regions, the crystal regions are more resistant to the enzyme hydrolysis. As a result, with the decrease of amorphous regions, the peaks of crystal lattices (101 and 002) become more and more prominent during the hydrolysis [49,50,51,52].

### 3.4. Effects of Enzymatic Hydrolysis on CrI and Average Crystal Size of Cellulose

**Figure 8:**
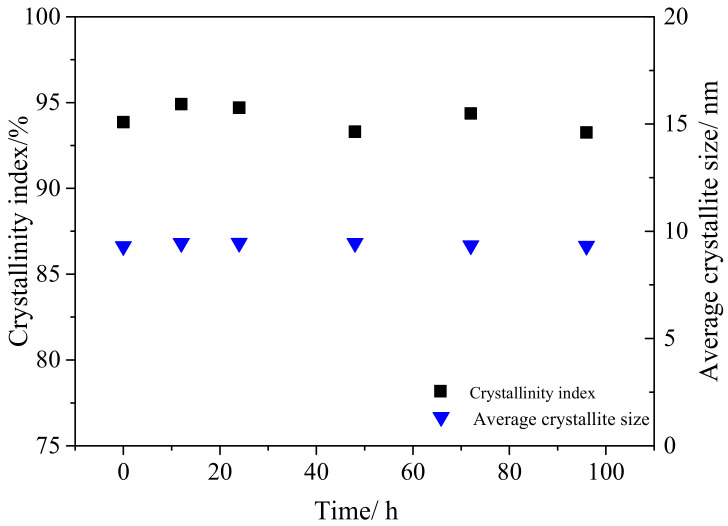
The variations of *CrI* and average crystallite size (*I_hkl_*) of tunicate cellulose during the enzymatic hydrolysis.

**Figure 9:**
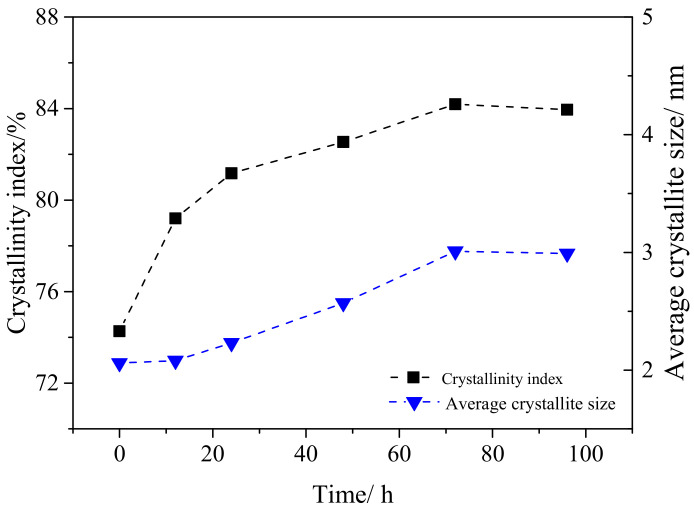
The variations of *CrI* and average crystallite size (*I_hkl_*) of α-cellulose during the enzymatic hydrolysis.

The *CrI* and average crystal size (*I_hkl_*) of tunicate cellulose and α-cellulose after enzymatic hydrolysis are represented in Figure 8 and Figure 9. During the enzymatic hydrolysis of, it was observed that the change of *CrI* for the tunicate cellulose (Figure 8) was little. The *CrI* fluctuated between 93.2% and 94.9%. Moreover, the crystalline sizes (*I_hkl_*) were almost the same. This is mainly due to its unique I*_β_* crystal cell structure: monoclinic cell with two chains, which render it a high density and make it thermodynamically and chemically stable [53,54,55].

Compared with the tunicate cellulose, the trend of *CrI* of α-cellulose (Figure 9) is totally different during the hydrolysis. The *CrI* increased first and then tended to be flat. The highest *CrI* (84.2%) was achieved at 72 h hydrolysis. As indicated in Figure 3, the α-cellulose consisted of amorphous and crystal regions in the cellulose structure. During the initial stage of hydrolysis, the amorphous region was likely to be degraded and hydrolyzed by the enzyme. On the contrary, the crystal region showed higher resistance to the enzyme attack [56]. As a result, the *CrI* decreased rapidly in the initial hydrolysis stage (0–24 h), following a slow decrease during the subsequent hydrolysis (24–96 h). Besides the *CrI*, the crystalline size of α-cellulose showed the same tendency as the *CrI*, indicating that the small cellulose crystals were vulnerable and were preferably hydrolyzed during the enzymatic hydrolysis. In addition, the crystal structure of α-cellulose is cellulose I*_α_* type. Compared with I*_β_* cellulose (monoclinic with two chains), I*_α_* cellulose has triclinic cell with one chain. This structure renders it a lower density and makes it less chemically stable [1].

### 3.5. Degree of Polymerization

Molecular weight (*M_w_*) and degree of polymerization (DP) of tunicate cellulose, α-cellulose and amorphous cellulose are represented in Table 2. Results indicate that similar *M_w_* (~2.48 × 10^6^ g/mol) and DPs (~4800) were exhibited during the hydrolysis. The variation between highest *Mw* and DP (appeared at 0 h) and the lowest *M_w_* and DP (appeared in 96 h) was only 1.04%. This tendency was in accordance with the *CrI* measurement in Figure 8. Again, thanks to its extremely high *CrI* and solid I*_β_* crystal lattice structure, the tunicate cellulose showed excellent ability of anti-biodegradation during the enzymatic hydrolysis. As for the case of α-cellulose, the values of *M_w_* and DP of α-cellulose are 1/6 time of tunicate cellulose. In addition, the *M_w_* and DP of α-cellulose were continuously decreased with the increase of hydrolysis time. Compared with the beginning of hydrolysis at 0 h, the *M_w_* and DP at 96 h were decreased by 20.8%. As discussed earlier, α-cellulose contained amorphous region, and its crystal lattice was I*_α_* type, which were both less resistant to enzymatic attack, compared with tunicate I*_α_* crystal lattice. As for the amorphous cellulose, the *M_w_* and DP decreased rapidly, by 27.9%, only after 7 h of hydrolysis. Furthermore, the amorphous cellulose was completely dissolved in the hydrolysis solution at that time. As a result, there were no *M_w_* and DP data for the amorphous cellulose after 7 h. In summary, during the enzymatic hydrolysis, the tunicate cellulose showed the highest, and the amorphous cellulose showed lowest, resistance to the biodegradation. The ability of α-cellulose was in between.

### 3.6. Cellulose Conversion Rate in Enzymatic Hydrolysis

**Figure 10:**
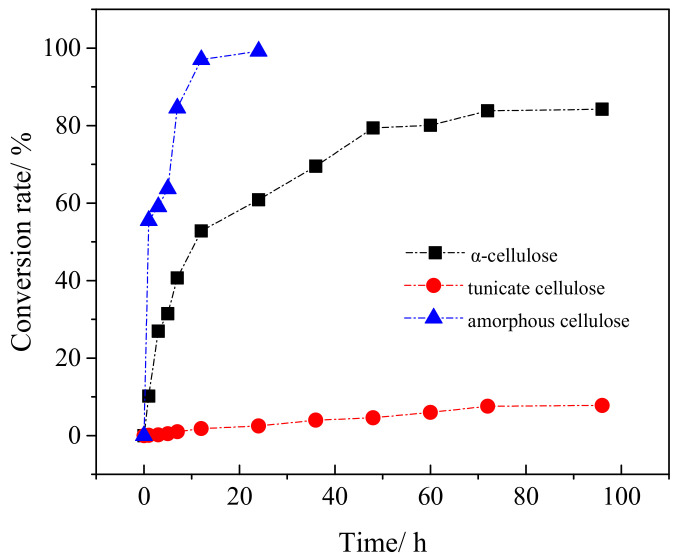
The cellulose conversion from various crystalline celluloses during enzymatic hydrolysis.

The cellulose conversion rate is the key parameter for evaluating enzymatic hydrolysis. Figure 10 shows the cellulose conversion rates in tunicate, α-cellulose and amorphous cellulose samples. Compared with α-cellulose and amorphous cellulose, the cellulose conversion rate of tunicate cellulose was extremely low. It was only 2.5% after 24 h of hydrolysis. After 96 h, the maximum sugar conversion just reached 7.8%, showing highly anti-enzymatic hydrolysis. However, for the case of α-cellulose and amorphous cellulose, the cellulose conversion rates were relatively fast during the initial stage. After 12 h, the rates were 52.8% and 97.0% for the α-cellulose and amorphous cellulose, respectively. As indicated in Figure 10, after 96 h, the majority of α-cellulose was hydrolyzed, and the cellulose conversion rate reached as high as 84.2%. However, the amorphous cellulose was completely hydrolyzed after 24 h. The variations of cellulose conversion rates between the tunicate, *α*- and amorphous celluloses are in agreement with the *Mw* and DP data in Table 1. The tunicate cellulose with high *CrI* had good stability and the highest ability of anti-enzymatic hydrolysis [57,58]. The most vulnerable one, amorphous cellulose, showed the lowest ability of anti-biodegradation, and it was completely hydrolyzed in a short period [59]. The ability of anti-biodegradation of the α-cellulose was in between the tunicate and pure amorphous cellulose, since it contained both the amorphous and crystal regions in the cellulose structure.

### 3.7. TEM Analysis of Cellulose Nanocrystals

**Figure 11:**
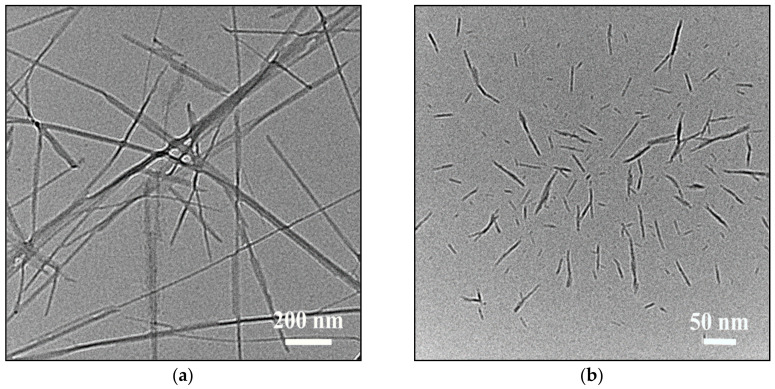
TEM images of (**a**) tunicate cellulose nanocrystals and (**b**) α-cellulose nanocrystal.

Figure 11 shows the TEM images of tunicate cellulose and α-cellulose nanocrystals. The length and width were calculated for tunicate nanocrystals (Figure 11a) and are 1.3 μm and 20 nm, respectively. The length-to-width ratio is 65, which is close to the literature data [3]. Similarly, the length and width calculated for α-cellulose nanocrystal (Figure 11b) are 300 nm and 20 nm, respectively, and the length-to-width ratio is 10.5, which is also close to the previous report [34]. From this morphology analysis, it is shown that the length-to-width ratio for tunicate cellulose is much higher than for α-cellulose, indicating its high mechanical strength [3,60].

### 3.8. Anti-Mold Effects of Tunicate and α-Cellulose Nanocrystals

As discussed earlier, in Section 2.2.6, in order to evenly disperse the tunicate and α-cellulose in the Martin agar medium, these two celluloses were hydrolyzed into nanocrystals by H_2_SO_4_, prior to the anti-mold experiment. Table 3 showed the variations of *CrI* of tunicate and α-celluloses before and after the acid hydrolysis. After the acid hydrolysis, the decrease of *CrI* was only 4.2% and 5.8% for the tunicate and α-celluloses, respectively. This indicated the acid hydrolysis had limit influences on the *CrI* of these two celluloses.

**Figure 12:**
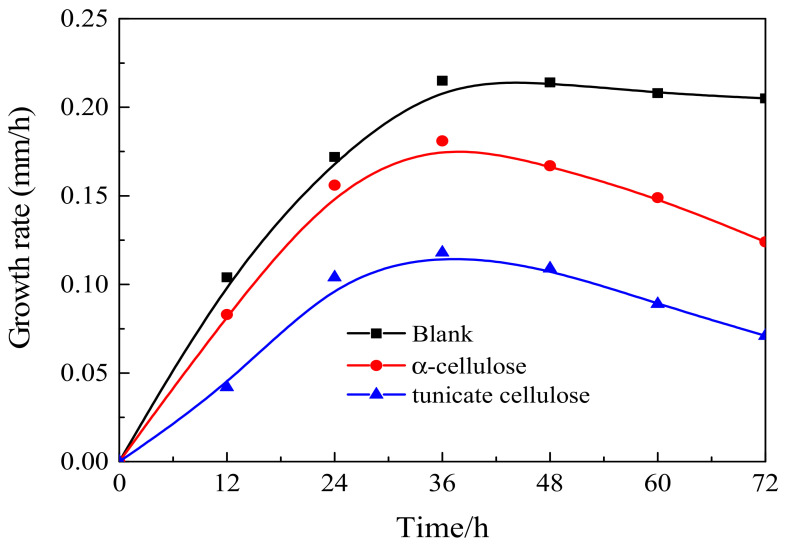
Anti-mold effect of tunicate cellulose nanocrystals and α-cellulose nanocrystals.

The mold growth rates of the blank sample, tunicate and α-cellulose nanocrystals are represented in Figure 12. It is clearly shown the mold growth rate in the blank sample was much higher than that of tunicate and α-celluloses. This was reasonable since the blank sample consisted of only the agar medium that was perfect for the mold cultivation. In addition, during the mold cultivation, the tunicate cellulose showed slower growth rate than the α-cellulose. At 36 h, the mold growth rate of tunicate cellulose was only 54.5% and 66.7% of the blank sample and the α-cellulose, respectively. The anti-mold effect of the tunicate cellulose was remarkable. This is due to the fact that the highly crystalized I*_β_* lattice in the tunicate cellulose has high anti-biodegrading abilities. However, the α-cellulose contained both the amorphous and I*_α_* crystal regions, which were less resistant to the bio-degradation, as compared with the tunicate cellulose. It should be noted that the mold growth rate was faster during the first 36 h of cultivation, and it was then followed by a gradual decrease in the subsequent experiment (Figure 12). This is due to the that, for the mold growth cycles, the mold grows fast and turns to mature in the first 36 h; then the mycelium growth gradually decreases. Consequently, the rate of mold growth reduced after 36 h of cultivation [61].

### 3.9. Measurement of Mold Growth Rate in the Medium Containing Various Amounts of Tunicate Nanocrystals

**Figure 13:**
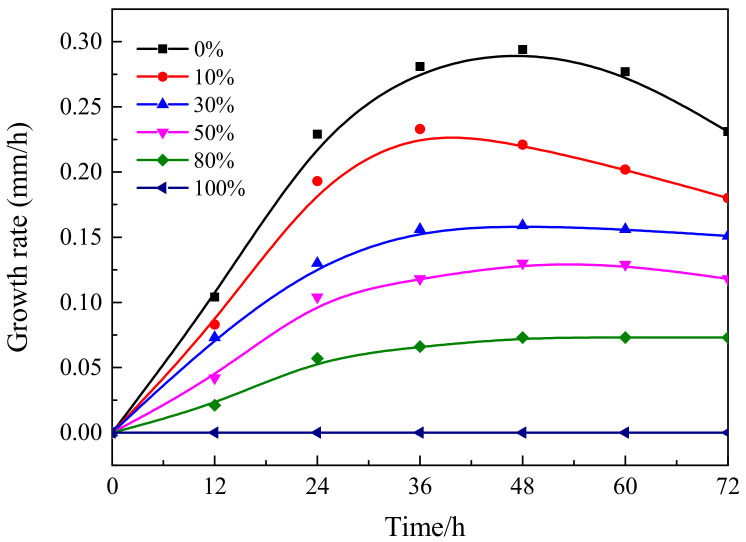
Antibacterial effect of different concentrations of tunicate cellulose nanocrystals.

Figure 13 shows the growth rate of mold in the medium containing various amounts of tunicate nanocrystals. The concentration of the tunicate nanocrystals used in this experiment was 1.4%. In the mold cultivation, the tunicate cellulose was mixed in Martin medium by different dry weights, i.e., 0%, 10%, 30%, 50%, 80% and 100%. As shown in Figure 13, with the increase of tunicate nanocrystals in the medium, the mold growth rate decreased significantly. At 36 h, the growth rate in the medium containing 80% of tunicate nanocrystals was 23.5% and 55.9% of the medium containing 10% and 50% of nanocrystals, respectively. It clearly shows that, the higher the content of tunicate nanocrystals in the medium, the slower mold growth rate during the cultivation. In addition, in the case that medium was only pure tunicate nanocrystals (medium containing 100% of tunicate nanocrystals), there was no trace of mold growth during the 96 h of cultivation. As a result, the mold growth rate line in the case was flat, and the value was zero. This finding indicated that the tunicate had excellent anti-mold ability, and this ability was proportional to its content in the medium. As discussed earlier in the paper, tunicate cellulose consists of almost-pure I*_β_* cellulose. The XRD measurement indicated that the crystallinity of tunicate cellulose is 93.9%. This significantly high crystallinity renders the tunicate cellulose high in anti-biodegradation to the micro-organisms, as shown in the enzymatic hydrolysis experiment and these mold cultivation tests. The anti-mold properties of tunicate nanocrystals might have great potentials in the manufacturing of anti-biodegradation package and biomedical materials.

## 4. Conclusions

Tunicate is different from other natural cellulose sources because it is the only animal species that generates cellulose in external tissues, contains a highly crystalized I*_β_* lattice and has good anti-biodegradation properties. In this research, we studied the enzymatic hydrolysis and anti-biodegradation properties of different types of celluloses, namely tunicate cellulose, α-cellulose and amorphous cellulose. Prepared tunicate cellulose was highly crystalline, had a large crystal size and had a high value of DP. XRD analysis exposed that the *CrI* of prepared tunicate cellulose and α-cellulose was 93.9% and 70.9%, respectively. Typical characteristic FTIR transmittance peaks of prepared different celluloses indicated that tunicate cellulose, α-cellulose and amorphous cellulose contained I*_β_*, I*_α_* and amorphous phase of cellulose, respectively. After enzymatic hydrolysis of cellulose with the cellulase enzyme, it was found that the crystal structure of tunicate cellulose builds a physical barrier to prevent the attack of enzymes, and after 96 h of hydrolysis, its crystal lattice was intact; however, for α-cellulose, the amorphous region was attacked by the enzyme. During enzymatic hydrolysis of tunicate cellulose, due to its unique monoclinic I*_β_* crystal cell structure, the *CrI* fluctuated between 93.2% and 94.9%, and the crystalline size (*I_hkl_*) was unchanged. The *M_w_* and DP are higher for tunicate cellulose, owing to its highly crystalline structure, which was almost unchanged during the enzymatic hydrolysis. However, amorphous cellulose was easily attacked by the enzyme, and after 7 h of hydrolysis, the DP decreased about 27.9%. It was further proved by cellulose conversion rate that the maximum cellulose conversion rate for tunicate cellulose and α-cellulose after 96 h enzymatic hydrolysis were 7.8% and 84.2%, respectively. After 12 h of hydrolysis, the amorphous cellulose was almost totally (97.0%) converted to sugar by the enzyme. TEM images proved that the length-to-width ratio of tunicate cellulose nanocrystals was higher (65) than α-cellulose nanocrystal (10.5), indicating high mechanical strength. Tunicate cellulose had excellent antibacterial ability, and its highly crystalized I*_β_* lattice strongly prohibited attacks from bacteria; as a result, the growth rate of mold was zero when the medium contained 100% tunicate cellulose nanocrystals. Due to its excellent resistance to micro-organisms, the tunicate cellulose could be potentially applied as renewable and anti-biodegradation materials.

## Figures and Tables

**Table 1:** XRD analysis of the cellulose crystallinity index (*CrI*) and crystallite size (*I_hkl_*).

Cellulose Sample	*CrI* (%)	*I_hkl_* Crystallite Size (nm)
Tunicate cellulose	93.9	9.3
α-cellulose	70.9	2.1
amorphous cellulose	-	-

**Table 2:** Molecular weight (*M_w_*) and polymerization degree of tunicate cellulose, α-cellulose and amorphous cellulose at different enzymatic hydrolysis stages.

Sample	Hydrolysis Time/h	*M_w_* (g/mol)	DP
tunicate cellulose	0	2.504 × 10^6^	4824
	12	2.493 × 10^6^	4803
	24	2.492 × 10^6^	4801
	48	2.489 × 10^6^	4795
	72	2.488 × 10^6^	4793
	96	2.478 × 10^6^	4774
α-cellulose	0	4.650 × 10^5^	896
	12	4.489 × 10^5^	865
	24	4.437 × 10^5^	855
	48	4.255 × 10^5^	820
	72	3.778 × 10^5^	728
	96	3.684 × 10^5^	710
amorphous cellulose	0	5.906 × 10^5^	1138
	1	5.475 × 10^5^	1055
	3	5.065 × 10^5^	976
	5	4.458 × 10^5^	859
	7	4.255 × 10^5^	820

DP, degree of polymerization.

**Table 3:** The variations of *CrI* of tunicate and α-celluloses before and after the acid hydrolysis.

	Before	After
Tunicate cellulose	93.9	90.0
α-cellulose	74.3	70.0
